# Nerves in the Tumor Microenvironment: Origin and Effects

**DOI:** 10.3389/fcell.2020.601738

**Published:** 2020-12-17

**Authors:** Wenjun Wang, Lingyu Li, Naifei Chen, Chao Niu, Zhi Li, Jifan Hu, Jiuwei Cui

**Affiliations:** ^1^Cancer Center, The First Hospital of Jilin University, Changchun, China; ^2^VA Palo Alto Health Care System and Stanford University Medical School, Palo Alto, CA, United States

**Keywords:** nerve, neurogenesis, cancer, perineural invasion, neurotrophins

## Abstract

Studies have reported the vital role of nerves in tumorigenesis and cancer progression. Nerves infiltrate the tumor microenvironment thereby enhancing cancer growth and metastasis. Perineural invasion, a process by which cancer cells invade the surrounding nerves, provides an alternative route for metastasis and generation of tumor-related pain. Moreover, central and sympathetic nervous system dysfunctions and psychological stress-induced hormone network disorders may influence the malignant progression of cancer through multiple mechanisms. This reciprocal interaction between nerves and cancer cells provides novel insights into the cellular and molecular bases of tumorigenesis. In addition, they point to the potential utility of anti-neurogenic therapies. This review describes the evolving cross-talk between nerves and cancer cells, thus uncovers potential therapeutic targets for cancer.

## Introduction

Globally, cancer is among the leading causes of mortality. Solid tumors are known to sculpt their microenvironment (e.g., molding angiogenesis pathways and forming pre-metastatic niches) in a bid to maximize their growth and metastatic potential (Magnon et al., 2013; Peinado et al., 2017). With regard to neurogenesis and angiogenesis processes, the process of cancer progression exhibits similarities with embryonic development, tissue repair, and regeneration (Boilly et al., 2017; Mauffrey et al., 2019). Neurons and nerve fibers have recently been identified as vital components of the tumor microenvironment that favor the initiation and progression of a variety of solid tumors (Stopczynski et al., 2014; March et al., 2020; Voronina et al., 2020). Accumulating evidence indicates that the nervous system participates in all stages of cancer, even in those that precede cancer development, such as pancreatic intraepithelial neoplasia (PanIN) or prostate intraepithelial neoplasia (PIN) (Magnon et al., 2013; Cole et al., 2015; Magnon, 2015; Saloman et al., 2016b; Kuol et al., 2018; Faulkner et al., 2019).

The nervous system regulates the functions of most organs. The peripheral nervous system (PNS), largely derived from the neural crest, connects the central nervous system (CNS) and the organs/limbs. The PNS consists of the motor and autonomic efferent fibers and sensory afferent fibers. The PNS and CNS mediate adaptive-reactive alterations in chronic diseases such as cancer. These alterations are referred to as “neuroplasticity” (Demir et al., 2015). “Neural plasticity” refers to the morphological and/or functional alterations of nerves, including changes in nerve fiber or trunk morphology, density, fiber qualities, and Schwann cell alterations (Demir et al., 2015). Studies have reported that the autonomic nervous system (ANS), composed of the sympathetic and parasympathetic divisions, plays important roles in tumorigenesis and cancer progression (Zhao et al., 2014; Rutledge et al., 2017). For example, gastric lesser curvature has higher vagal innervation, larger ganglia, and a higher incidence of gastric cancer when compared to the greater curvature (Zhao et al., 2014). Patients with spinal cord injuries have lower incidences of prostate cancer (PCa) due to functional denervation of the prostate gland indicating nerve dependence in PCa (Rutledge et al., 2017). The nervous system also indirectly influences cancer progression by regulating hormone secretion, such as epinephrine [E] and cortisol, through the hypothalamic-pituitary-adrenal (HPA) axis. The involvement of sensory neurons in tumorigenesis and cancer progression has also been reported (Saloman et al., 2016a). Besides modulating cancer proliferation and metastasis, the nervous system also regulates different aspects of cancer such as inflammation and angiogenesis (Stopczynski et al., 2014). In turn, immune cells or stromal cells can also be involved in nerve dependence in cancer.

Generally, cancer neurobiology has important implications in cancer pathogenesis and therapy. In this review, we elucidate on the crosstalk between nerves and cancer cells as well as the role of stress in cancer. Therefore, we present potential avenues for exploiting the emerging roles of the aforementioned processes in cancer prognosis and treatment.

## Nerve Emergence in the Tumor Microenvironment

**FIGURE 1:**
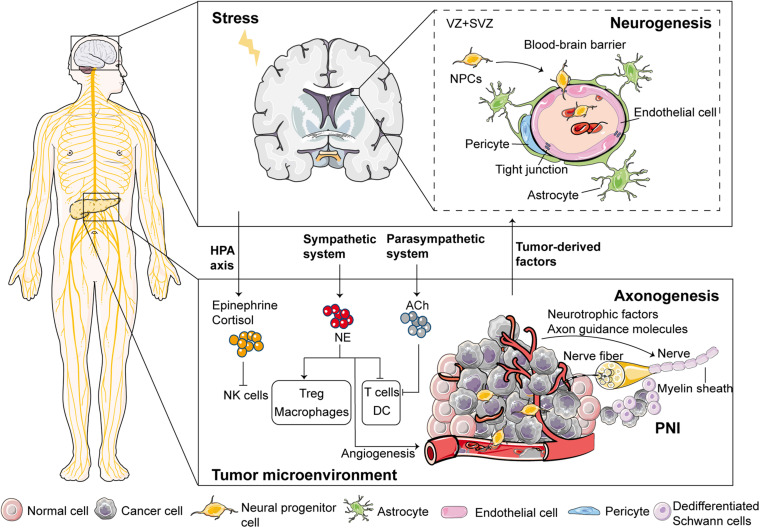
Proposed mechanisms involved in the emergence of nerves in the tumor microenvironment. Regarding neurogenesis (i.e., increased number of neurons), the neural progenitor cells (NPCs) leave the subventricular zone (SVZ) and egress into circulation through the disrupted blood-brain barrier during cancer development. After reaching and infiltrating the primary tumor or metastatic tissues, these NPCs differentiate into new adrenergic neurons, which regulate tumor growth and metastasis. For axonogenesis (i.e., tumor-induced neural sprouting toward the tumor microenvironment), cancer cells and some stromal cells can enhance neuronal outgrowth and initiate tumor innervation through multiple mechanisms. Sympathetic nerves release NE while parasympathetic nerves release ACh in the tumor microenvironment, and are, therefore, involved in modulating immune responses. Moreover, stress regulates immune responses through two major neural pathways; the HPA axis and the sympathetic nervous system. Schwann cells can act as leading cells that actively dissociate, recruit, and guide cancer cells to neurites through heterocellular contact, thereby, leading to PNI. VZ, ventricular zones.

Apart from blood and lymphatic vessels, evidence indicates that neurogenesis (increased number of neurons) and axonogenesis (tumor-induced neural sprouting toward the tumor microenvironment) also play a vital role in tumorigenesis and cancer progression. Studies have reported that neurogenesis and axonogenesis are present in pre-neoplastic lesions and probably contribute to the initiation of cancer as an early event of the pre-malignant phase (Ayala et al., 2008; Mauffrey et al., 2019; Figure 1).

### Stem Cell Neurogenesis

Neurogenesis involves the proliferation and differentiation of neural stem cells (NSCs) (Lois and Alvarez-Buylla, 1994). It generates neural progenitor cells (NPCs) with high migration abilities, followed by erminal differentiation of the NPCs into mature neural cells (Lois and Alvarez-Buylla, 1994). Neurogenesis majorly occurs during embryonic and early postnatal brain development (Lois and Alvarez-Buylla, 1994). It has been identified in several human brain areas after birth, with the generated neurons often migrating long distances (up to several centimeters) from the ventricular zones (VZ)/sub-ventricular zone (SVZ) before reaching their final destination (García-González et al., 2017). NSCs and NPCs persist throughout life in SVZ of the lateral ventricles and the subgranule zone of the dentate gyrus in the hippocampus (Venkatesh and Monje, 2017). Studies have reported that NSCs and NPCs can generate new neurons and glia throughout life (Lois and Alvarez-Buylla, 1994).

A retrospective case-controlled and age-matched study found that the number of neurons was higher in prostatic ganglia in patients with PCa than in controls (Ayala et al., 2008). This result supports the existence of neurogenesis in PCa (Ayala et al., 2008). A possible hypothesis for the origin of cancer-related neo-neurons could be that; stem cells give rise to cells that differentiate into neurons (Mauffrey et al., 2019). Mechanisms that drive neurogenesis in tumors can be compared to those that underlie neural degeneration-regeneration (Mauffrey et al., 2019). Neural progenitors proliferate in the CNS after brain injury or during tissue regeneration to generate newborn neurons which migrate to injured sites to ensure a functional wound-healing response. The Na-K-Cl cotransporter 1 (NKCC1) has been shown to regulate cell volume (Mejia-Gervacio et al., 2011). It also helps maintain the migratory speed of neuroblasts along the rostral migratory stream (Mejia-Gervacio et al., 2011). Recent studies have suggested that NPCs [doublecortin (DCX) +] are transported from the CNS (SVZ) into circulation after blood-brain barrier (BBB) disruption during cancer development (Mauffrey et al., 2019). DCX is a microtubule-associated protein that is principally expressed in immature migrating neurons (Brown et al., 2003). It is one of the most important markers of migrating neuroblasts (Brown et al., 2003). After reaching and infiltrating primary tumors or metastatic tissues, these NPCs can further differentiate into new adrenergic neurons that are involved in PCa growth and metastasis (Mauffrey et al., 2019).

However, the upstream signaling pathways that contribute to the occurrence of neurogenesis in cancer have not been elucidated (García-González et al., 2017). Besides, questions on how neural progenitors penetrate the BBB, and thus migrate from the SVZ and what regulates different stages of neural progenitor differentiation in tumors have not been answered. The BBB comprises a complex array of tight junctions formed by endothelial cells, pericytes, astroglia, and perivascular mast cells (Rozniecki et al., 2010). Acute stress activates perivascular brain mast cells through the release of corticotropin-releasing-hormone which then disrupt the BBB (Rozniecki et al., 2010). Postnatal SVZ neurogenesis is modulated by multiple neurotransmitters (small molecules released by synapse or synapse-like structures) such as serotonin, acetylcholine (ACh), and dopamine (Venkatesh and Monje, 2017). The migratory process of NPCs in response to brain damage is regulated by multiple mechanisms in the tumor microenvironment, including: cellular pathway activation (e.g., DCX and PI3K/Akt); receptor-mediated interactions and adhesion molecules (e.g., chemokines); and cellular junction as well as extracellular matrix (ECM)-related proteins [e.g., matrix metalloproteinases (MMPs)] (Zarco et al., 2019). For example, postnatal neuroblasts express serotonin receptor 3A (5HT3A) and serotonergic modulation plays a role in postnatal migratory streams that originate from the SVZ (García-González et al., 2017). Notably, serotonin and the serotonin receptor are dysregulated during metastasis and angiogenesis in several cancers (Alpini et al., 2008; Sarrouilhe and Mesnil, 2019). Cholangiocarcinoma patients show high levels of serotonin due to elevated expression of tryptophan hydroxylase 1 (the rate-limiting enzyme in the biosynthesis of serotonin), and the suppressed expression of monoamine oxidase A (an enzyme accounting for degradation of serotonin) (Alpini et al., 2008). Future studies should aim at investigating the underlying mechanisms of stem cell neurogenesis in cancer patients, and whether the mechanism of neurogenesis is the same across different cancers.

### Axonogenesis

Internal organs and glands are regulated by PNS, especially ANS. For instance, the stomach is predominantly innervated by the parasympathetic nervous system while the pancreas is regulated by both sympathetic and parasympathetic nerves. Most solid tumors (except the brain and spinal cord tumors) are innervated by nerve fibers that arise from the PNS, which form part of the tumor microenvironment. It is postulated that alterations in neurotrophic factor signaling before tumorigenesis can impact the progression from precancerous lesions to cancer by influencing tumor precursor cells and/or tissue innervation (Ayala et al., 2008). In a study involving genetically engineered pancreatic ductal adenocarcinoma (PDAC) mice models, alterations in neurotrophic factor expression and their receptors (*Ngf, Gfr*α*2*, and *Nrtn*) in the pancreas were shown to occur during the premalignant phase (Stopczynski et al., 2014). Moreover, PanIN lesions were caused by hyper-innervation in neurotrophic factor-rich microenvironments (Stopczynski et al., 2014). It is worth noting that axonogenesis in cancer may overlap with axonogenesis that occurs during embryonic development, where neurotrophic growth factors released from organs drive axonogenesis.

**FIGURE 2:**
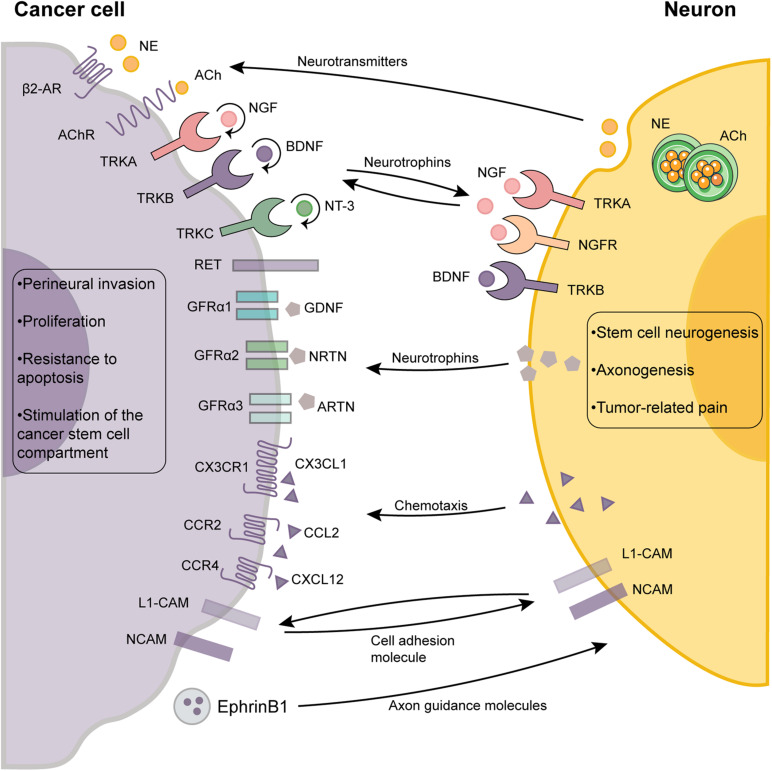
The cross-talk between cancer cells and neurons in the tumor microenvironment. The reciprocal autocrine and paracrine signaling between the cancer cells and neurons plays a pivotal role in neurogenesis, axonogenesis and perineural invasion. Autocrine signaling (round arrows) of cancer cells also promotes tumor growth and dissemination by activating multiple signaling pathways. The outcome of the interactions between neurons and cancer cells are shown as boxes. ACh, acetylcholine; β2-AR, β2-adrenergic receptors; BDNF, brain-derived neurotrophic factor; GDNF, glial cell line-derived neurotrophic factor; GFRα, glial cell line-derived family receptor alpha; L1-CAM, L1 cell adhesion molecule; NCAM, neural cell adhesion molecule; NGF, neurotrophins composing of nerve growth factor; NT-3, neurotrophin-3; NE, norepinephrine; TRK, tyrosine receptor kinase.

Nerve fibers that innervate normal tissues can be chemoattracted to tumor environments and outgrow as a result of neurotrophic factors that are released by the cancer cells. Cancer cells can promote neuronal outgrowth and initiate their own innervation through paracrine secretion, a phenomenon referred to as the neurotrophic effect (Figure 2). Neurotrophins composed of nerve growth factor (NGF), brain-derived neurotrophic factor (BDNF), neurotrophin-3 (NT-3), and NT-4/5 have been shown to drive axonogenesis by stimulating tyrosine kinase receptors expressed in nerve terminals. To exert biological effects, neurotrophic factors bind the tyrosine receptor kinase (TRK) family [also called neurotrophic tyrosine kinase receptors (NTRKs)], including TRKA, TRKB, and TRKC and the p75 neurotrophin receptor (p75^NTR^) (Chao, 2003; Arévalo and Wu, 2006; Lagadec et al., 2009). The two families are categorized as cell membrane receptors where the TRK family represents a specific high-affinity receptor whereas p75^NTR^ is considered the low-affinity nerve growth receptor (NGFR) (Arévalo and Wu, 2006).

The roles of NGF and its receptors (TRKA and p75^NTR^) in cancer are well established (Laurent et al., 2004; Voronina et al., 2020). Breast cancer cells can stimulate neuronal growth using multiple neurotrophins such as NGF which can be released by Ca2^+^-mediated exocytosis of NGF-containing vesicles (Laurent et al., 2004; Voronina et al., 2020). NGF can also be secreted by tumor-associated immune cells and fibroblasts. Similarly, neuronal differentiation and axonogenesis can be induced by the proNGF (the precursor of NGF), which was shown to be secreted by PCa cells *in vitro* (Pundavela et al., 2014). In ovarian cancer, there is a feedforward loop through which adrenergic signaling can induce BDNF secretion from tumor cells via a β-adrenergic receptor 3 (ADRβ3)/cAMP/Epac/JNK-dependent manner. The BDNF/TRKB signaling axis further upregulates tumoral innervation (Allen et al., 2018). Immunohistochemical analysis revealed significantly elevated expression levels of BDNF and TRKB in hepatocellular carcinoma (HCC) (Guo et al., 2011).

Additionally, axon guidance molecules such as netrins, secreted by cancer cells act in synergy with neurotrophic growth factors to enhance axonogenesis (Biankin et al., 2012; Madeo et al., 2018). During the early pancreatic cancer stages, some axon guidance genes, especially those encoding for proteins of the SLIT/ROBO signaling pathway, exhibited recurrent mutations and copy-number variations (Biankin et al., 2012). Dysregulated axon guidance genes were also identified in murine models of early pancreatic carcinogenesis, suggesting that they are involved in its pathogenesis (Biankin et al., 2012). Similarly, evidence from other cancers including lung, breast, kidney, and cervical cancer has also implicated aberrant SLIT/ROBO signaling in carcinogenesis. Cancer cells secrete an exosome that packages the axonal guidance molecule, EphrinB1, which was shown to induce axonogenesis *in vitro* and tumor innervation *in vivo* (Madeo et al., 2018). Therefore, the crosstalk between cancer cells and nerve cells largely contributes to axonogenesis. However, more studies are required to characterize other mediators.

## The Effects of Nerves on Tumors

The nervous system has been reported to be a regulator of cancer development and progression in multiple tissues (Magnon et al., 2013; Cole et al., 2015; Magnon, 2015; Saloman et al., 2016b; Kuol et al., 2018; Faulkner et al., 2019). In addition to being direct stimulators of cancer cells, nerves also exert a broad and extended impact on the tumor microenvironment by establishing interactions with other cells such as stromal, endothelial, and immune cells in the tumor microenvironment (Zahalka et al., 2017).

### Nerves Regulate the Local Inflammation and Immune Response

The perineural inflammatory response is attributed to various inflammatory mediators that are locally released by afferent neurons (Stopczynski et al., 2014; Saloman et al., 2016a). Chronic inflammation increases the risk of malignancy and cancer progression (Stopczynski et al., 2014). Even a few sensitized pancreatic afferents can induce neurogenic inflammation in early pre-tumor stages, thereby contributing to, or accelerating the progression from PanIN to PDAC (Stopczynski et al., 2014). Neonatal capsaicin treatment can be used to ablate the sensory neuron that innervates the pancreas (Saloman et al., 2016a). This procedure can prevent neurogenic inflammation and prolong the survival of PDAC mice in a dose-dependent manner (Saloman et al., 2016a).

Neural regulation of immune responses has been identified as an emerging field in cancer biology (Dantzer, 2018). Vagal immunomodulatory effects, also known as the cholinergic anti-inflammatory pathway, have been linked to the action of acetylcholine, which is the predominant neurotransmitter of the parasympathetic system. Vagotomy elevates the risk of, or mortality from gastric, colorectal, and lung cancer (Åhsberg et al., 2009). This elevation has been attributed to the inhibition of anti-inflammation (Åhsberg et al., 2009). The nicotinic acetylcholine receptor-α7 (α7nAChR) is widely expressed in various immune cells, such as lymphocytes, suggesting that the vagus nerve may be involved in regulating immune responses in the tumor microenvironment (Antonica et al., 1994). Sectioning the right vagus was shown to reduce the number of lymphocytes released from the thymus into venous circulation (Antonica et al., 1994). The vagus nerve can also upregulate the expression of *Tff2*, which encodes a secreted anti-inflammatory peptide in memory T cells, thereby, suppressing the expansion of myeloid-derived suppressor cells in the spleen as well as cancer development (Dubeykovskaya et al., 2016). Nicotine, a selective cholinergic agonist, inhibits the transcriptional activity of nuclear factor-κB and the production of proinflammatory mediators in human peripheral monocytes using α7nAChR (Yoshikawa et al., 2006).

In contrast, the sympathetic nervous system (SNS) modulates β-adrenergic receptors (β-AR) signaling of immune cells in tumors through the circulating epinephrine/NE or local NE secretion by SNS nerve fibers (Nissen et al., 2018). Innate immune cells express ADRα1, ADRα2, and ADRβ2 while adaptive immune cells mainly express ADRβ2 (Dantzer, 2018). Studies have reported that the activity of innate immune cells is modulated by activation of ARs (Huan et al., 2017). The SNS activates ADRα1 to enhance the activity of Kupffer cells (i.e., macrophages in the liver) in regulating inflammatory responses, thereby accelerating HCC occurrence (Huan et al., 2017). Through ADRβ2, epinephrine induces macrophage polarization toward the M2 type that accelerates breast cancer progression (Qin et al., 2015). AR signaling can also inhibit the maturation, migration, and antigen presentation of dendritic cells (DC) (Ueshima et al., 2013). Glucocorticoids enhance the expression of ADRβ2 in natural killer (NK) cells and suppress the number and cytotoxic activity of NK and NKT cells, thereby inhibiting tumor immunosurveillance (Inbar et al., 2011; De Lorenzo et al., 2015). With regards to adaptive immunity, activation of the β-AR signaling pathway significantly suppresses CD8^+^ T cells, including their production, proliferation, and cytolytic killing capacity of IFN-γ. It also activates regulatory T cells (Tregs) (Nissen et al., 2018). Activation of the β2-AR signaling pathway in lymphocytes modulates the responsiveness of retention-promoting chemokine receptors CCR7 and CXCR4, thereby blocking the egress of lymphocytes from lymph nodes (Nakai et al., 2014). On the contrary, a recent study demonstrated that the ablation of SNS contributes to the immature myeloid-derived suppressor cells (MDSCs) accumulation, which facilitates the expansion of Tregs and suppresses tumor immunity (Nevin et al., 2020). In all, targeting the communication between neurons and leukocytes may be a potential approach for alleviating immunosuppression in the tumor microenvironment.

### Perineural Invasion (PNI) Is a Vital Route of Solid Tumor Dissemination

Perineural invasion (PNI) refers to the invasion of the perineurium of large nerves that surround the tumor by cancer cells, thus causing them to metastasize. Generally, PNI is a frequently disregarded route of solid tumor dissemination, except for the direct invasion of surrounding tissues, lymphatics, and haematogenic spread (Marchesi et al., 2010b). For example, some head and neck neoplasm are in anatomical proximity with some cranial nerves, which can serve as channels with low resistance for migration (Liu et al., 2020). However, recent studies have considered PNI pathogenesis to be an active invasion of cancer cells into the perineurium space rather than a simple passive diffusion (Marchesi et al., 2010b). PNI is common among tumors of highly innervated organs such as pancreatic cancer and PCa. PDAC cells have been demonstrated to exist inside celiac ganglion and its surrounding, suggesting a fashion of tumor spread along the sensory afferents or the sympathetic nerves that synapse in the celiac ganglion (Stopczynski et al., 2014).

The peripheral nerves are made up of three layers (the endoneurium, the perineurium, and the epineurium) (Amit et al., 2015). The endoneurium encloses several nerve fibers, which are composed of an axonal process wrapped with a myelin sheath supported by Schwann cells (Amit et al., 2015). Despite the varying incidences of PNI in different cancers, it is associated with poor prognosis and reduced survival (Bapat et al., 2011). Results from a retrospective study that enrolled 133 extrahepatic cholangiocarcinoma patients revealed that 73.7% of the patients had PNI (Murakami et al., 2013). Those patients exhibited a significantly low overall 5-year survival rate (Murakami et al., 2013). Intraneural invasion (i.e., the invasion of cancer cells in the endoneurium) exerts a worse impact on clinical prognosis than PNI (Amit et al., 2015). However, the mechanisms by which cancer cells destroy the peripheral nerve barrier have not been established.

PNI is mediated by various signaling molecules secreted by cancer cells and nerves (Bapat et al., 2011). Nerve fibers can secrete multiple active neuropeptides and neurotransmitters within the vicinity of cancer or stromal cells to activate corresponding membrane receptors involved in tumor growth and metastasis (Li et al., 2014; Table 1).

**TABLE 1:** Molecular factors involved in perineural invasion (PNI).

**Category**	**Molecule**	**Effects in PNI**	**References**
Neurotransmitters and their receptors	NE/ADR	Promote cancer cells proliferation, migration, and PNI	Guo et al., 2013; Ma et al., 2019
	ACh/AChR	Provoke proliferation and migration of cancer cells	March et al., 2020
Neurotrophins and their receptors	NGF/TRKA/p75^*NTR*^	Mediate a mutual tropism between the nerves and cancer cells	Ceyhan et al., 2008; Ma et al., 2008
	NT-3/TRKC	Unclear	—
	NT-4/5	Unclear	—
	BDNF/TRKB	Promote cancer cell proliferation	Sclabas et al., 2005
	GDNF/RET/GFRα	Facilitate migration and PNI	Veit et al., 2004; Gil et al., 2010
Chemokines and their receptors	CX3CR1/CX3CL1	Contribute to migration and PNI	Marchesi et al., 2010a
	CCR2/CCL2		He et al., 2015
	CXCR4/CXCL12		Zhang et al., 2008
MMPs	MMP-2	Degrade the matrix around the tumor and the nerve tissue	Klein et al., 2004
	MMP-9		
Other cell-surface molecules and receptors	L1-CAM	A membrane receptor of the immunoglobulin supergene family; contribute to PNI and neuropathic pain	Yamanaka et al., 2007; Ben et al., 2010; Na’ara et al., 2019
	NCAM	A membrane receptor of the immunoglobulin supergene family that regulates neuronal adhesion and migration	Kameda et al., 1999; Shang et al., 2007
	Semaphorin 3A/plexins A1-A4 and neuropilin-1	Regulate the interactions between nerves and cancer cells in the tumor microenvironment	Müller et al., 2007
	Semaphorin 4F		Ding et al., 2013

*ACh, acetylcholine; ADR, adrenergic receptors; AChR, acetylcholine receptor; BDNF, brain-derived neurotrophic factor; CX3CL1, C-X3-C motif chemokine ligand 1; CX3CR1, C-X3-C motif chemokine receptor 1; CCL2, C-C chemokine ligand 2; CCR2, C-C chemokine receptor 2; CXCR4, C-X-C chemokine receptor type 4; CXCL12, C-X-C motif chemokine 12; GDNF, glial cell line-derived neurotrophic factor; GFRα, GDNF family receptor-alpha; L1-CAM, L1 cell adhesion molecule; MMPs, matrix metalloproteinases; NCAM, neural cell adhesion molecule; NE, norepinephrine; NGF, nerve growth factor; NT-3, neurotrophin-3; NT-4, neurotrophin-4; PNI, perineural invasion; p75^*NTR*^, p75 neurotrophin receptor; RET, rearranged during transfection; TRKA, tropomyosin receptor kinase A; TRKB, Tropomyosin receptor kinase B; TRKC, tropomyosin receptor kinase C.*

#### Neurotransmitters and Their Receptors in PNI

Several neurotransmitters have been reported to be highly correlated with PNI (Ma et al., 2019; March et al., 2020). Catecholamines are neurochemicals that have catechins and amines converted from tyrosine as their precursor. They include norepinephrine (NE), epinephrine, and dopamine. NE/β2-AR signaling promotes proliferation, migration, and PNI by inducing epithelial-mesenchymal transition (EMT) and upregulating MMPs (MMP-2 and MMP-9) in salivary adenoid cystic carcinoma (Ma et al., 2019). NE has also been shown to accelerate pancreatic cancer PNI and progression through the β-AR/PKA/STAT3 signaling pathway *in vivo*, where the activation of STAT3 upregulates the downstream expression levels of NGF and MMPs (Guo et al., 2013). Parasympathetic nerves provoke the proliferation and migration of PCa cells by secreting Ach, which stimulates cholinergic receptor muscarinic 1 (March et al., 2020). In addition, monoamine oxidase A (MAOA), a catecholamine neurotransmitter degrading enzyme, was found to be remarkably decreased by epigenetic alterations in clinical HCC samples (Li et al., 2014). Therefore, MAOA downregulation is closely associated with metastasis and poor prognoses among HCC patients (Li et al., 2014).

#### Neurotrophins and Their Receptors in PNI

Several studies have supported the involvement of neurotrophic factors in PNI (Sclabas et al., 2005; Ma et al., 2008; Wang et al., 2009). A positive correlation was observed among the expression levels of NGF, p75^NTR^, TRKA, TRKB, glial cell line-derived neurotrophic factor (GDNF), and PNI in pancreatic cancer tissues or cell lines (Sclabas et al., 2005; Ma et al., 2008; Wang et al., 2009). However, NGF was found to have no correlation with PNI, recurrence-free, and OS in extrahepatic cholangiocarcinoma (Urabe et al., 2016). Dorsal root ganglion (DRG) and pancreatic cancer cell co-culture models suggested that a mutual tropism exists between the nerves/DRGs and pancreatic cancer cells through multiple molecular factors (e.g., NGF-TRKA pathway) (Ceyhan et al., 2008; Ma et al., 2008). Besides, TRKB signaling induces EMT, resulting in elevated cancer cell migration and metastasis (Fujikawa et al., 2012). However, the involvement of NT-3/4 in PNI has not yet been established.

The GDNF family of ligands (GFL) comprises four neurotrophic factors, including GDNF, neurturin (NRTN), artemin (ARTN), and persephin (PSPN) (Airaksinen and Saarma, 2002). GFLs bind co-receptors of the glial cell line-derived family receptor alpha (GFRα) family (GDNF to GFRα1, NRTN to GFRα2, ARTN to GFRα3, and PSPN to GFRα), followed by the recruitment and activation of transmembrane RET receptor tyrosine kinase (Airaksinen and Saarma, 2002). GDNF can be secreted by nerves, Schwann cells, and endoneurial macrophages. It was shown to recruit pancreatic cancer cells expressing RET and GFRα1 in both co-culture and mice models (Gil et al., 2010; Cavel et al., 2012). It also facilitates the migration and PNI of PDAC cells through the PI3k/Akt and Ras-Raf-MEK-ERK pathways (Veit et al., 2004; Ben et al., 2010). The NRTN/GFRα-2 axis also elevates neural density and an aggressive phenotype in pancreatic cancer (Wang et al., 2014).

#### Chemokines and Their Receptors in PNI

Chemokines are a class of small soluble proteins that modulate cell migration during wound healing, embryonic development, and tumor metastasis (Marchesi et al., 2010a; He et al., 2015). CCL2, a chemokine released by DRG/nerve, contributes to migration and PNI of PCa cells that express its receptor (CCR2) (He et al., 2015). Apart from being a chemokine, CCL2 can also act as a repair factor that is secreted after neural injury caused by cancer infiltration during a nerve repair inflammatory response (He et al., 2015). In addition, the CX3CL1 chemokine was found to be expressed by neurons while its receptor, CX3CR1, was overexpressed in both PDAC cell lines and surgical samples (Marchesi et al., 2010a). Therefore, the CX3CR1/CX3CL1 axis plays a role in PNI and dissemination of pancreatic cancer cells along intra- and extra-pancreatic nerves (Marchesi et al., 2010a). CXCL12/CXCR4 has been shown to stimulate the invasiveness of PCa cells *in vitro*, and increase the number of nerves *in vivo* (Zhang et al., 2008).

#### Other Cell-Surface Molecules or Receptors in PNI

The L1 cell adhesion molecule (L1-CAM, also known as CD171) and neural cell adhesion molecule (NCAM, also known as CD56) are members of the neuronal immunoglobulin superfamily that regulates neuronal adhesion and migration (Ben et al., 2010). Peripheral nerve injury induces the upregulation of L1-CAM in the contact surface of injured peripheral axons and Schwann cells, suggesting its possible involvement in nerve regeneration (Yamanaka et al., 2007). A recent study reported that Schwann cells secret L1-CAM, which acts as a chemo-attractant, thus aiding in the recruitment of cancer cells by activating the MAPK signaling pathway (Na’ara et al., 2019). L1-CAM also elevates the expression levels of MMPs, including MMP-2 and MMP-9, by activating STAT3 in PDAC cells (Na’ara et al., 2019). The main role of MMP-2 and MMP-9 in PNI is degrading the ECM around the tumor and nerve tissue (Klein et al., 2004). Therefore, overexpression of L1-CAM and NCAM has significant correlations with PNI, vascular invasion, and poor overall survival outcomes among cancer patients (Kameda et al., 1999; Shang et al., 2007; Ben et al., 2010).

Semaphorins are a family of proteins that act as axon guidance factors during cancer development (Müller et al., 2007; Ding et al., 2013). Plexins are their main functional receptors. Semaphorin 3A and its receptors, plexins A1-A4 and neuropilin-1 (NRP1), are overexpressed in pancreatic cancer and are correlated with negative clinicopathological manifestations (Müller et al., 2007). Cytoplasmic expression of semaphorin 4F in PCa cells is positively correlated with nerve density, PNI diameter, and PCa recurrence risk (Ding et al., 2013).

#### The Roles of Schwann Cells in PNI

Schwann cells are peripheral glial cells that are involved in neural homeostasis, repair, and regeneration. Their emergence has been detected in murine and human PanIN lesions (Demir et al., 2014). They are chemoattracted to cancer cells and their precursor lesions by NFG-p75^NTR^/TRKA, suggesting that they migrate first in the premalignant phase (Bentley and Lee, 2000; Demir et al., 2014). P75^NTR–/–^ mice embryos exhibited a deficit in Schwann cell migration (Bentley and Lee, 2000).

Cancer cells take advantage of the canonical functions of Schwann cells to promote PNI (Deborde et al., 2016). For example, invagination of Schwann cells between individual axons during human fetal development separates them from each other, thereby isolating individual axons from large bundles. Schwann cells induce protrusions in cancer cells and intercalate between cancer cells to facilitate their dispersal from the tumor (Deborde et al., 2016). In addition, during nerve repair, Schwann cells dedifferentiate/activate, lose their myelination ability, induce and guide axonal extensions. This is followed by the re-expression of some proteins, such as glial fibrillary acidic protein (GFAP) (reviewed by Jessen and Mirsky, 2016). At the same time, there is a significant increase in the number of GFAP-positive Schwann cells when cancer cells injure/invade nerves (Deborde et al., 2016). Schwann cells can also guide cancer cells toward neurites by inducing the extension of directed protrusions in cancer cells (Deborde et al., 2016). Therefore, they can act as leading cells that actively dissociate, recruit, and guide cancer cells to neurites through heterocellular contact that ultimately leads to PNI (Deborde et al., 2016).

### Nerves Sustain Tumor Proliferation and Induce Resistance to Apoptosis

Autocrine secretion of neurotrophins modulates the growth and dissemination of cancer cells by activating different signaling pathways. Pro-survival signals can be triggered by NGF binding to TRKA; BDNF and NT-4 binding to TRKB; and NT-3 binding to TRKC. Studies have reported that breast and pancreatic cancer cells express receptors such as TRKA and phospho-TRKA for several neuropeptides and neurotransmitters (Lagadec et al., 2009). Sclabas et al. (2005) reported that 50% of the human pancreatic cancer specimens in their study had overexpressed TRKB levels (Sclabas et al., 2005). TRKA can be activated in an autocrine manner since the majority of breast cancers express NGF (Adriaenssens et al., 2008; Lagadec et al., 2009). Expression of NGF stimulates tumor cell proliferation and survival through the constitutive activation of the PI3K-Akt and ERK/p38 MAP kinase (MAPK) pathways (Adriaenssens et al., 2008; Lagadec et al., 2009). In addition, neuronal endings have been shown to secrete a soluble synaptic protein, neuroligin-3 (NLGN3), which stimulates high-grade glioma proliferation and further NLGN3 expression through the PI3K-mTOR signaling pathway (Venkatesh et al., 2015). The proliferation of gastric cancer cells can be accelerated by Netrin-1 through the ERK/MAPK signaling pathway and focal adhesion kinase (FAK) activation (Yin et al., 2018).

Most of the cytotoxic anti-cancer drugs can trigger the p53-dependent apoptotic program of cancer cells. Studies have shown that catecholamines mediate the chemotherapeutic resistance of cervical cancer cells which overexpress ADRβ2 both *in vitro* and *in vivo* (Chen et al., 2017). Catecholamines elevate the expression levels of the silent information regulator 1 (Sirt1), a type III histone deacetylase, by activating β2-adrenergic receptor signaling (Chen et al., 2017). Therefore, they can inhibit doxorubicin-induced p53-dependent cytotoxicity in cervical cancer cells since Sirt1 impairs p53 functions by mediating its deacetylation (Chen et al., 2017).

Anoikis refers to programmed cell death that is associated with losses in cell-matrix interactions. Metastatic success of tumor cells is dependent on their ability to resist anoikis (Douma et al., 2004; Fujikawa et al., 2012; Okugawa et al., 2013). Overexpressed TRKB enhances tumor metastasis by suppressing caspase-associated anoikis (Douma et al., 2004; Fujikawa et al., 2012; Okugawa et al., 2013). In contrast, TRKC is considered to be a tumor suppressor and a good-prognostic factor since it induces apoptosis in the absence of ligands such as NT-3 (Bouzas-Rodriguez et al., 2010). Upregulated autocrine NT-3 secretion has been reported in most of the aggressive human neuroblastomas (NBs). It blocks TRKC-induced apoptosis of NB cell lines (Bouzas-Rodriguez et al., 2010).

### Tumor Innervation Drives Tumor Angiogenesis

Generally, nerves bundle along blood vessels. They can provide a critical set of signals that assist tumors to redevelop a vascular network, which ensures nutrition and communication during cancer proliferation and progression (Zahalka et al., 2017). Studies have documented the roles of neurotransmitters and neuropeptides in regulating angiogenesis (Kermani et al., 2005; Zahalka et al., 2017).

A study using mice models reported that adrenergic nerves stimulate angiogenesis in early PCa growth by the release of NE (Zahalka et al., 2017). The study indicated that angiogenesis requires an alteration of endothelial cell metabolism from oxidative phosphorylation to aerobic glycolysis (Zahalka et al., 2017). In addition, adrenergic signals from autonomic nerves fuel angiogenesis and cancer progression by inhibiting oxidative phosphorylation in endothelial cells (Zahalka et al., 2017). In breast cancer cell lines, direct activation of β-AR signaling was shown to enhance the expression of proangiogenic factors, including vascular endothelial growth factor (VEGF) and interleukin (IL)-6 (Madden et al., 2011). Neuropeptide Y, a sympathetic neurotransmitter, stimulates VEGF expression, thereby promoting angiogenesis and breast cancer progression in a paracrine manner *in vitro* (Medeiros and Jackson, 2013). Nicotine can also accelerate the proliferation of endothelial cells as well as angiogenesis through nAChR especially α7nAChR (Pillai and Chellappan, 2012). BDNF recruits bone-marrow derived Sca-1 + hematopoietic cell subsets expressing TRKB to enhance capillary formation (Kermani et al., 2005). By binding to the plexin B1 receptor on endothelial cells, semaphorin 4D, secreted by the tumor-associated macrophages (TAMs), accelerates tumor angiogenesis and vessel maturation (Sierra et al., 2008).

### Nerves Stimulate the Cancer Stem Cell (CSC) Compartment

Accumulating evidence indicates that innervation impacts the development of stem cell compartments and tissue homeostasis (Zhao et al., 2014; Yin et al., 2015; Hayakawa et al., 2017). Recent studies have indicated a similar crosstalk between nerves and CSC. The intrinsic regulation of CSCs occurs through vital proliferative and survival pathways such as the Wnt, Notch, and Hedgehog pathways (Yin et al., 2015). Cholinergic nerves can activate Wnt signaling in gastric stem cells through muscarinic receptor-3 (M3R), which activates yes-associated protein (YAP). This activation contributes to stem cell expansion (Zhao et al., 2014; Hayakawa et al., 2017). However, vagal nerve signaling exhibits an opposite effect that inhibits *KRAS*-mutated PDAC (Renz et al., 2018). Cholinergic signaling, directly and indirectly, suppresses CSC compartment and PDAC cell growth partly through the cholinergic receptor muscarinic 1 (CHRM1). This leads to the inhibition of the downstream MAPK/EGFR and PI3K/AKT pathways (Renz et al., 2018). Sensory nerves activate Hedgehog signaling in touch dome epithelia, an innervated subset of interfollicular epidermis cells, thereby conferring high susceptibilities for the occurrence of basal cell carcinomas (Peterson et al., 2015).

Paracrine BDNF of differentiated recurrent triple-negative breast cancer (TNBC) cells promote the self-renewal potential of TRKB^+^ CSC through transcriptional activation of KLF4 (Yin et al., 2015). These TRKB^+^ cells represent a distinct CSC subpopulation, accounting for recurrent TNBC growth (Yin et al., 2015). In head and neck squamous cell carcinoma (HNSCC), p75^NTR^ is considered to be a more specific marker of CSCs than CD44 (Murillo-Sauca et al., 2014). However, the CSC-regulating potential for nerves does not rule out their indirect roles, such as interactions with the immune system.

### Nerves Are Involved in the Formation of Tumor-Related Pain

Pain, a typical cancer sign, impacts on the quality of life and functional status of cancer patients (Jimenez-Andrade et al., 2011). Several mechanisms, including inflammation, tumor-induced acidosis, and tumor-induced injury to peripheral nerve fibers are associated with the generation of tumor-related pain (Peters et al., 2005; Jimenez-Andrade et al., 2011; Cohnen et al., 2020).

Inflammatory cells such as proinflammatory macrophages and dendritic cells are recruited by cancer cells to the outside of the sciatic nerve. They initiate a series of events, including epineurium damage and depletion of triglycerides from epineural adipocytes (Cohnen et al., 2020). Jimenez-Andrade et al. suggested that normal nerve fibers that innervate tissues can be sensitized or injured by factors that are released by cancer and tumor-associated stromal/inflammatory/immune cells (Jimenez-Andrade et al., 2011). Sensitized nerve fibers are vulnerable to noxious stimuli and tumor-induced and/or released factors such as NGF, endothelins, prostaglandins, protons, bradykinin, and cytokines (Jimenez-Andrade et al., 2011). Emerging evidence has revealed that the NGF signaling pathway involves inflammatory hyperalgesia and cancer-associated pain (Mizumura and Murase, 2015). Besides, NGF interacts with TRKA and/or p75^NTR^ expressed on the perineurium, thereby, directly activating and sensitizing sensory nerves that are close to pancreatic cancer. NGF also regulates the expression and function of transient receptor potential vanilloid 1 (TRPV1), a non-selective cation channel, thereby, stimulating and activating sensory neurons (Zhu et al., 2011).

PNI causes prominent cancer pain, such as severe back pain in PDAC patients. Many of the PNI signaling molecules overlap with those in pain signaling (Sclabas et al., 2005; Bapat et al., 2011). Moreover, invading cancer cells can damage the neuronal sheath (Sclabas et al., 2005; Bapat et al., 2011). PNI of the head and neck cancer can result in cranial neuropathy, such as trigeminal neuropathy, which is characterized as facial or paresthesias pain (Boerman et al., 1999). In PDAC, pancreatic nerve hypertrophy and PNI are associated with PDAC-related pain. This pain is attributed to nerve damage by the invading tumor cells, and sensitized pancreatic afferents (Stopczynski et al., 2014). L1-CAM might also be involved in the occurrence of neuropathic pain by activating intracellular signaling cascades, such as p38 MAPK in nociceptive pathways (Yamanaka et al., 2007).

## Stress Promotes Cancer Progression

The nervous system can perceive signals from the outside environment, process them, and transmit them to the CNS, where they are then integrated. Studies have shown that cancer patients frequently suffer from chronic emotional stress due to the social, emotional, and physical effects caused by cancer (Chida et al., 2008). Epidemiological studies have established an association between chronic stress conditions and high risk or poor survival outcomes among cancer patients (Lillberg et al., 2003; Chida et al., 2008). Some viable explanations account for the link between psychological stress and tumorigenesis. For example, people under stress are more likely to develop some unhealthy behaviors (e.g., excessive drinking, smoking, etc.) or a long-term inflammatory response, which raise the risk for tumorigenesis (Dai et al., 2020). Stress affects various immunological and neurochemical aspects involved in tumorigenesis and cancer progression (Thaker et al., 2006; Feng et al., 2012; Le et al., 2016; Zhou et al., 2016). It regulates immune responses through two major neural pathways; the HPA axis and the SNS pathways (Reiche et al., 2004).

Under stress conditions, activation of the ANS or the HPA axis leads to the secretion of diverse mediators (e.g., glucocorticoids and catecholamines) that provoke tumorigenesis and cancer progression (Thaker et al., 2006; Zhou et al., 2016). Chronic stress was shown to promote tumorigenesis in mice partially by attenuating p53 function (Feng et al., 2012). During chronic stress, elevated glucocorticoid levels induce a negative regulator of p53, the serum- and glucocorticoid-induced protein kinase (SGK1), to suppress p53 function (Feng et al., 2012). In a stressed mouse model, elevated levels of tissue catecholamines were shown to activate ADRβ2, which upregulated *VEGF* gene expression in ovarian carcinoma cells, resulting in enhanced angiogenesis and malignant cell growth (Thaker et al., 2006). The TAMs in ovarian cancer patients express elevated VEGF and MMP-9 because of the high psychological stress levels (e.g., chronic life stress, depression, and perceived stress) (Lutgendorf et al., 2008). Since macrophages express β-adrenergic and α-adrenergic receptors, the mechanisms underlying these associations are attributed to elevated stress hormones, including NE and cortisol (Lutgendorf et al., 2008). Similarly, elevated NE levels enhance the invasive potential of ovarian cancer cells most likely due to higher expression levels of MMP-2 and MMP-9 (Sood et al., 2006). Chronic stress can induce the proliferation of hematopoietic stem cells as well as increased levels of inflammatory leukocytes including neutrophils and inflammatory monocytes (Heidt et al., 2014). Mechanically, elevated NE levels decrease CXCL12 through ADRβ3 on bone marrow niche cells (Heidt et al., 2014). CXCL12 is known to restrain the proliferation and migration of the hematopoietic stem and progenitor cell (Heidt et al., 2014). This is reflected from the side, the immunosuppressive activity induced by corticosteroid hormones in the HPA axis seem to not play a major role in stress. On the positive side, social support can alleviate such a phenomenon (Lutgendorf et al., 2008).

Surgical stress upregulates epinephrine and NE secretion, which bind βAR in immune cells (Zhou et al., 2016). Elevated epinephrine markedly promotes Treg proliferation, which suppresses antitumor immunity in breast cancer patients (Zhou et al., 2016). However, during the perioperative period, propranolol, a non-selective β-adrenergic antagonist, has been reported to restore cell-mediated immunity in breast cancer patients with surgery-induced immunosuppression (Zhou et al., 2016). In addition, cold stress was shown to suppress antitumor immune responses and accelerated tumor growth and metastasis in mice (Kokolus et al., 2013). However, Walker et al. reported that in the mice received denervation of the splanchnic nerves, stress-enhanced cancer proliferation and metastasis were not affected by elimination of circulating epinephrine (Walker et al., 2019). In all, the activities of catecholamines may vary from cancer to cancer by binding different target cells.

A previous study indicated that chronic stress in mice elevated lymphatic vessel density, a stronger flow in lymphatic vessels, and dilation to promote tumor cell dissemination (Le et al., 2016). Activation of TAMs by the β-adrenoceptor signaling pathway enhances their ability to secrete enhanced inflammatory molecules (e.g., PGE2), which stimulate the tumor cells to secrete VEGFC that is necessary for lymphatic remodeling (Le et al., 2016). Targeting SNS can inhibit the effect of chronic stress on lymphatic remodeling to prevent tumor cell dissemination through the lymphatic routes (Le et al., 2016). Therefore, psycho-behavioral intervention or therapies for stress and anxiety disorders might synergistically improve the efficacy of cancer treatment.

## Clinical Value and Application

A better understanding of cancer neurobiology is necessary as it provides new insights into the prognosis and therapeutic targets for tumors.

### Nerves Are an Emerging Hallmark of Cancer

Neurogenesis and axonogenesis are emerging as a feature of aggressive tumors, such as colorectal cancer (Albo et al., 2011). Assessment of nerve density in the tumor microenvironment might serve as a potential predictive marker of cancer aggressiveness. A retrospective study involving radical prostatectomy tissues from 43 prostate adenocarcinoma patients revealed that the densities of sympathetic and parasympathetic nerve fibers in the tumor and surrounding normal tissues are correlated with poor clinical outcomes (Magnon et al., 2013).

PNI, as an underestimated route of metastasis, has an association with poor prognosis or recurrence (Yamaguchi et al., 2002). The neoplastic cells disseminating along the nerve fascicles probably escape from resection (Chatterjee et al., 2012; Murakami et al., 2013; Zhou et al., 2015). In PNI-positive gastric cancer patients, the largest involved nerves (with a diameter ≥ 65 μm) were correlated with low 5-year disease-free survival outcomes (Zhou et al., 2015). However, a retrospective study involving 186 patients with parotid gland malignancies concluded that PNI positivity does not have a statistically significant correlation with worse OS and disease-free survival (Huyett et al., 2018). Moreover, facial nerve paresis and anesthesia were both significant symptoms for predicting PNI of parotid gland malignancies (Huyett et al., 2018). PNI assessment is usually based on the histological evaluation of tissue sections or full-body imaging, such as MRI (Boerman et al., 1999). Advanced microscopy techniques and imaging techniques can reveal fine structural analysis of neuron-cancer cell interactions in multiple tissue types of clinical samples (Chung et al., 2013).

Neurotransmitters (including epinephrine and NE), neurotrophins, as well as their receptors have been identified as potential diagnostic and prognostic cancer biomarkers (Abdel-Hamid et al., 2016). Overexpressed proNGF has been documented to be a clinical biomarker that predicts the prognosis of human PCa, since it correlates with the Gleason score (Pundavela et al., 2014). Upregulated TRKB expression level has been proposed to be an independent prognostic marker for gastric cancer (Okugawa et al., 2013).

### Potential Anti-neurogenic Therapies Are Promising in Cancer Treatment

Multiple effects of nerves in the tumor microenvironment have been documented. Therefore, tumor innervation is emerging as an optional therapeutic target (Table 2). Since PNI has dual effects in tumor metastasis (regulate relapse after surgical resection and cancer pain generation) it is a promising therapeutic target.

**TABLE 2:** Therapeutic perspectives in cancer neurobiology.

**Targets**	**Drug**	**Type of cancer**	**Beneficial effects**	**Stage of development**	**References or clinical trial number**
NGF	Anti-NGF sequestering Ab (mAb911)	Bone sarcoma	Reduce neuropathic pain; Relieve tumor-induced bone destruction	Preclinical	McCaffrey et al., 2014
	Anti-NGF Ab or small interfering RNA against NGF	Breast cancer	Inhibit tumor growth and metastasis	Preclinical	Adriaenssens et al., 2008; Hondermarck, 2012
	Anti-NGF sequestering Ab	Prostate cancer	Relieve cancer-induced bone pain	Preclinical	Jimenez-Andrade et al., 2011
	Anti-NGF Ab (Tanezumab)	Bone metastases	—	Phase 2	NCT00830180
TRK	Small molecule inhibitor (AZ64) that targets TRKB	HNSCC	Overcome chemotherapy resistance	Preclinical	Yilmaz et al., 2010
	TRK antagonist (K252a)	Gastric cancer; choriocarcinoma	Inhibit tumor growth and invasion	Preclinical	Kawamura et al., 2010; Okugawa et al., 2013
	Oral TRK inhibitor (CEP-701)	Advanced carcinomas	—	Phase 1	Marshall et al., 2005
	An Oral TRKA/TRKB/TRKC/ROS1/ALK inhibitor (Entrectinib/RXDX-101)	Locally advanced or metastatic cancer; solid tumors; primary brain tumors	—	Phase 1	NCT02097810
				Phase 2	NCT02568267
				Phase 1/2	NCT02650401
	A highly selective pan-TRK inhibitor (NOV1601/CHC2014)	Solid tumors	—	Phase 1	NCT04014257
	Oral TRKA inhibitor (VMD-928)	Advanced solid tumors or lymphoma	—	Phase 1	NCT03556228
	A small molecule inhibitor of TRKA/TRKB/TRKC/ALK (TSR-011)	Advanced solid tumors or lymphoma	—	Phase 1/2	NCT02048488
	An Oral ROS1 and TRK inhibitor (DS-6051b)	Solid tumors	—	Phase 1	NCT02279433
	A specific inhibitor of TRKA, B, and C (AstraZeneca 1332)	PDAC	—	Preclinical	Johnson et al., 2017
βAR	β-blockers	PCa	Reduce PCa-specific mortality	Preclinical	(Grytli et al., 2014)
	β-blockers (propranolol, propranolol hydrochloride)	Breast, pancreatic, colorectal, cervical, gastrointestinal cancer; melanoma; PCa; locally recurrent/metastatic solid tumors	—	—	NCT00502684
			Phase 2	NCT02650401	
			Phase 2	NCT03838029	
			Phase 2	NCT03919461	
			—	NCT01988831	
			Phase 2	NCT01902966	
			Phase 2	NCT02944201 NCT03152786	
			Early Phase 1	NCT03245554	
			Early Phase 1	NCT02013492	
Nerve fibers	Botulinum toxin	Gastric cancer	—	Phase 2	NCT01822210

#### Denervation

From a therapeutic perspective, based on the critical roles of nerves in cancer development, surgical or pharmacological denervation (the injection of neurotoxic drugs) constitutes a potential and innovative strategy for preventing cancer dissemination, improving survival, or inhibiting pain (Zhao et al., 2014). The unilateral vagotomy and injection of botulinum toxin A have been reported to attenuate gastric tumorigenesis and progression (Zhao et al., 2014). A phase 2 clinical trial evaluated the effect of botulinum toxin injection by gastroscopy in both the tumor and the adjacent stomach wall tissues of gastric cancer patients (NCT01822210). Such denervation, combined with other therapies is a viable approach for cancer patients who do not accept surgery as a treatment option. However, determining the timing of this treatment and coping with the corresponding side effects is very critical. For instance, denervation has been shown to promote the development of cancer-related lesions in the gastric remnant (Kaminishi et al., 1997).

#### Antagonists of Receptors

In the clinic, β-blockers have been extensively used to treat patients with hypertension, angina, or anxiety (Dézsi and Szentes, 2017). β-blockades have been shown to increase cancer patient survival, though the specific mechanism has not been established (Hayakawa et al., 2017; Kokolus et al., 2018). A combination of inhibiting angiogenesis through both neural signals and/or endothelial cell metabolism is a novel approach for overcoming antiangiogenic resistance (Thaker et al., 2006; Zahalka et al., 2017). A retrospective study involving 3561 PCa patients with high-risk or metastatic disease indicated that β-blockers reduce PCa-specific mortality (Grytli et al., 2014). Targeting β-AR signaling has been suggested to work in tandem with cancer immunotherapy to enhance therapeutic responses (Kokolus et al., 2018; Nissen et al., 2018). Various clinical trials are assessing the efficacy of β-blockers (e.g., propranolol, propranolol hydrochloride) for the treatment of prostate (NCT02944201 and NCT03152786), colorectal (NCT03919461), breast (NCT02596867), gastrointestinal (NCT03245554), pancreatic (NCT03838029) cancers, and melanoma (NCT01988831) as well as locally recurrent or metastatic solid tumors (NCT02013492). Propranolol has been tested in combination with neoadjuvant chemotherapy in breast (NCT01847001) and gastric (NCT04005365) cancer patients. Studies encompassing diverse tumor types should be done to evaluate the efficacy of β-blockades as adjuvant therapies in clinical oncology. Adrenergic signaling-induced immunosuppression may necessitate a combination of immunotherapies with β-blockers to improve efficiency.

#### Inhibitors of Neuroendocrine Factors

Targeting neurotrophins and neurotrophic signaling has been shown to suppress tumor progression and cancer-associated pain (Adriaenssens et al., 2008; McCaffrey et al., 2014; Hayakawa et al., 2017). Anti-NGF antibodies or small interfering RNAs against NGF inhibited breast tumor growth and metastasis in xenograft models (Adriaenssens et al., 2008). Given the impact of NGF in cancer pain, NGF blockade inhibits the sprouting of sensory nerve fibers and alleviates cancer-associated pain, especially bone cancer pain, through anti-hyperalgesia (i.e., normalizing a downregulated nociceptive threshold) (Bloom et al., 2011; Jimenez-Andrade et al., 2011; Mizumura and Murase, 2015). The efficacy of blocking antibodies against NGF (e.g., tanezumab, fasinumab) in patients with pain or cancer-induced pain have been investigated in some clinical trials (NCT00830180) (Mizumura and Murase, 2015). NGF blockade at early bone sarcoma stages has been shown to relieve tumor-induced bone destruction as well as reduce pain by 40–70% (McCaffrey et al., 2014). Therefore, inhibiting NGF could play a dual effect against both cancer growth and cancer-associated pain.

Furthermore, given the role of TRKs in tumor cell proliferation and PNI, inhibiting RTKs has therapeutic significance (Yilmaz et al., 2010). In HNSCC, a potent and selective TRK kinase inhibitor, AZ-64 that targets TRKB, has been shown to inhibit tumor proliferation and migration, as well as overcome chemotherapy resistance *in vitro* (Yilmaz et al., 2010). The TRK antagonist, K252a, was reported to inhibit the growth and invasion of gastric cancer as well as choriocarcinoma *in vivo* and *in vitro* (Kawamura et al., 2010; Okugawa et al., 2013). Several clinical trials are investigating the therapeutic potential of entrectinib, which is an orally bioavailable TRKA/B/C, ROS1, and ALK inhibitor in multiple tumors (NCT02097810, NCT02568267, and NCT02650401). In addition, several TRK inhibitors, including NOV1601 (NCT04014257), VMD-928 (NCT03556228), TSR-011 (NCT02048488), and DS-6051b (NCT02279433), are being evaluated in the on-going clinical trials. However, the results of these studies have not yet been reported. Combined with radiotherapy, AstraZeneca 1332, an inhibitor for TRKA, B, and C was reported to have an inhibitory effect on pancreatic cancer growth *in vitro*, while it exhibited inefficient effects in xenograft growth (Johnson et al., 2017).

## Future Directions

In conclusion, the reciprocal signaling between cancer cells and nerves provides novel insights into the mechanisms of tumorigenesis and cancer progression. It has not been established whether neurogenesis and axonogenesis are coincidental or prerequisites in cancer development and progression. Moreover, it is unknown if these processes are specific to different cancer types. Studies have described the phenomenon of autonomic or sensory nerves in cancer. However, the potential involvement of motor nerves in cancer cannot be ignored and further investigations are recommended. Different cancer types utilizing various molecules or targeting to varied nerve types results in either the promotion or suppression of cancer development. More studies should explore the precise cellular and molecular mechanisms that underlie tumor innervation and PNI. However, PNI can only be evaluated after surgery, thus necessitating the need to establish predictive biomarkers for the existence or severity of PNI. It makes sense for surgeons to preoperatively assess the existence of PNI, carefully, and cope with the resection border during surgery. The development and implementation of single-cell sequencing and multi-omics assays may provide new insights into rare cells such as NPCs in the tumor microenvironment. Manipulation of the nervous system presents a potential avenue for the establishment of prognostic markers, risk stratification, and therapeutic strategies (including adjuvant treatment of cancer-induced pain) for cancer. However, many questions have yet to be answered. Different nerves function at different times and in different ways. For example, sympathetic nerves facilitate the early stages of carcinogenesis, while parasympathetic nerves accelerate cancer dissemination (Magnon et al., 2013). Denervation is considered to be most effective during the early cancer stages, even before the precancerous stage (Saloman et al., 2016b). Given these factors, what determines the intervention (e.g., denervation) time and who a suitable patient is? Further experimental or clinical studies should be performed to investigate the translational value or clinical applications of cancer neurobiology.

## Author Contributions

WW and LL carried out the primary literature search, drafted and revised the manuscript, and participated in discussions. NC, CN, and ZL helped to modify the manuscript. JC and JH carried out the design of the research and literature analysis, drafted and revised the manuscript, and participated in discussions. All authors read and approved the final manuscript.

## Conflict of Interest

The authors declare that the research was conducted in the absence of any commercial or financial relationships that could be construed as a potential conflict of interest.

## Abbreviations

Ab: antibody
ALK: anaplastic lymphoma kinase
β-AR: β-adrenergic receptors
mAb: monoclonal antibody
NGF: nerve growth factor
HNSCC: head and neck squamous cell carcinoma
PCa: prostate cancer
PDAC: pancreatic ductal adenocarcinoma
ROS1: ROS proto-oncogene 1
TRK: tyrosine receptor kinase.
